# Supplementary material for: Flowering Time Variation in Two Sympatric Tree Species Contributes to Avoid Competition for Pollinator Services
Source: Plants (Basel). 2023 Sep 22;12(19):3347. doi: 10.3390/plants12193347 (PMC10574496; doi:10.3390/plants12193347)
Supplement: Supplementary file 1 [file plants-12-03347-s001.zip › plants-2541415-supplementary.pdf]

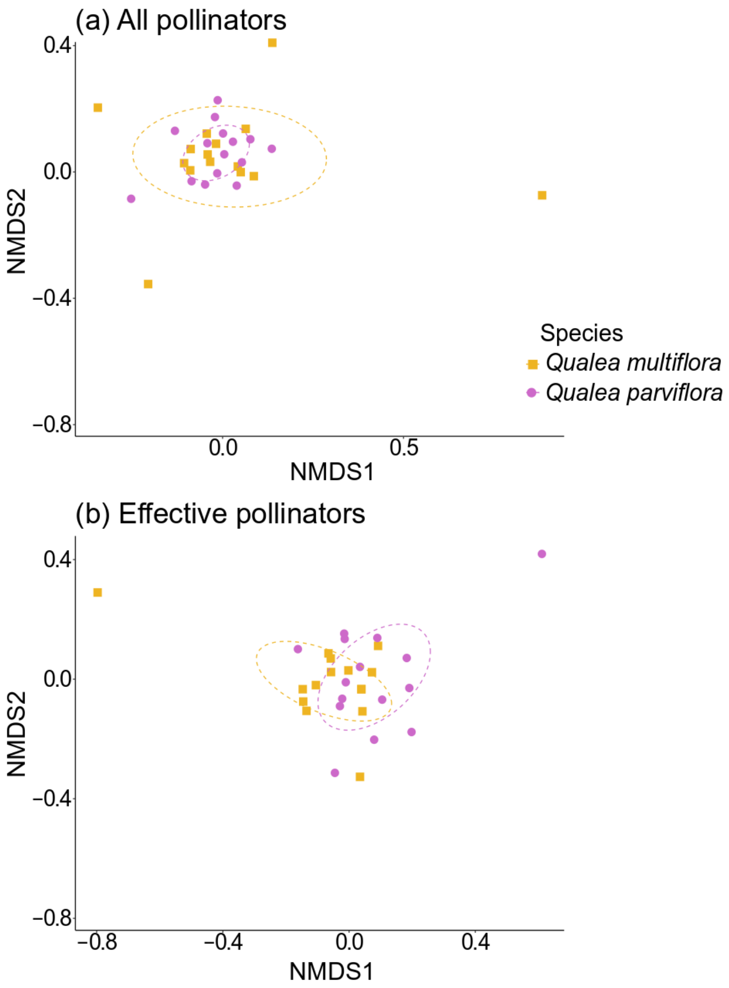

Figure S1. Composition of similar pollinators' between *Qualea multiflora* and *Q. parviflora.* All pollinators (ANOSIM=-0.011; p=0.518; stress=0.154) (a) only effective pollinators (ANOSIM=-0.009; p=0.514; stress=0.176) (b). Dots represent plants of *Qualea multiflora* and *Q. parviflora*. Ellipses represent the 95% confidence interval.

Table S1. Niche overlapping analysis comparing all pollinators, effective pollinators, floral scent and flowering phenology in *Qualea multiflora* and *Q. parviflora*. Flowering did not present values for null model (RA4) because there were no overlap patterns, and the algorithm does not support the analysis. CZ – Czekanowski metric.

	Coefficient overlap			Null model (RA3)				Null model (RA4)			
	Dhat1	*Pianka*	*CZ*	*Observed index*	*Simulated index*	*p-value*	*SES (Standardized Effect Size)*	*Observed index*	*Simulated index*	*p-value*	*SES (Standardized Effect Size)*
All pollinators	0.736	0.872	0.718	0.872	0.613	<0.001	2.862	0.872	0.751	0.015	2.004
Effective pollinators	0.752	0.877	0.749	0.877	0.462	<0.001	3.147	0.877	0.720	0.027	1.948
Scent	0.421	0.565	0.333	0.565	0.356	0.002	3.155	0.565	0.395	0.003	3.099
Flowering	0.004	0	0	0	0.187	1	-0.780	-	-	-	-

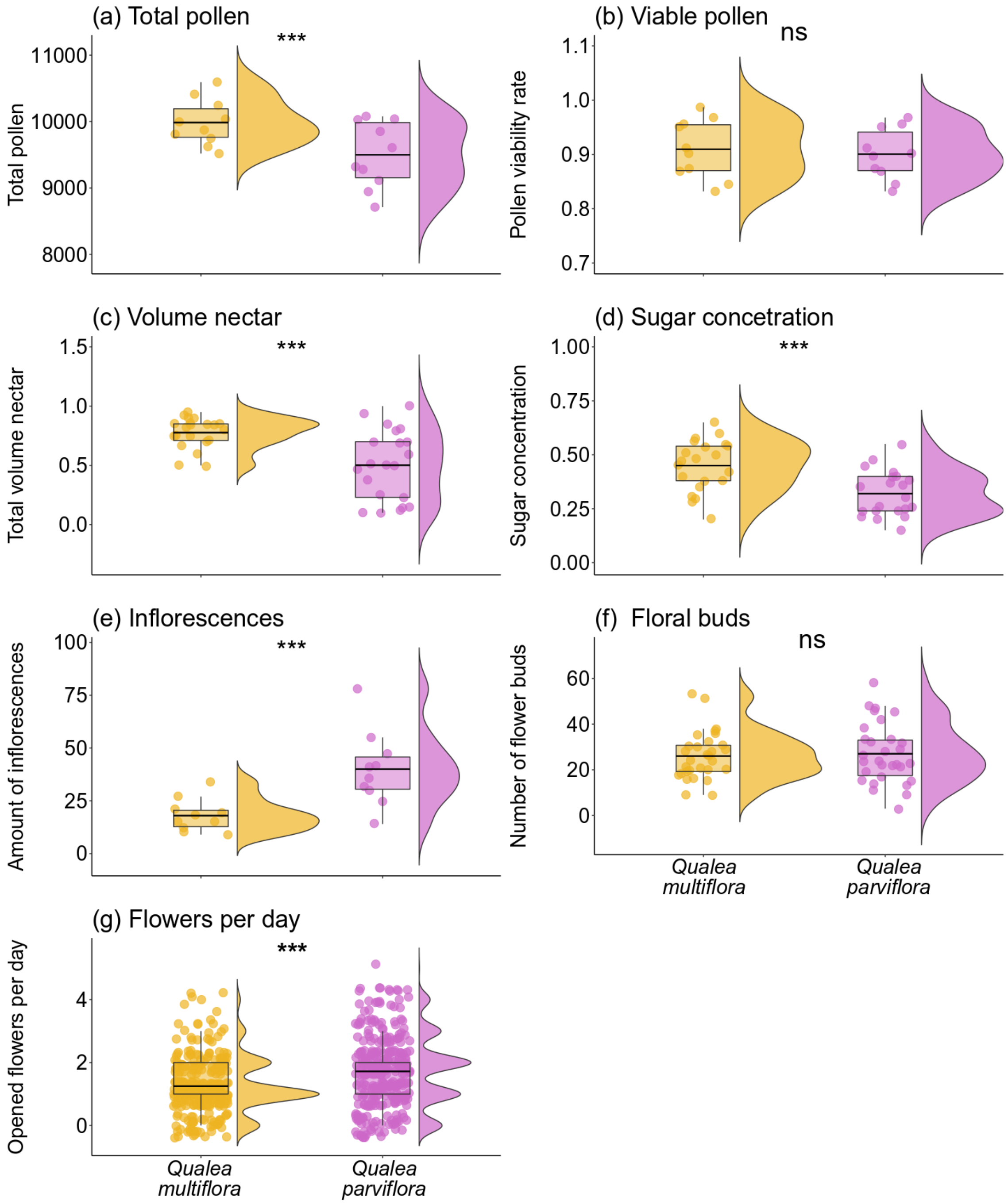

Figure S2. Comparison of pollen number (a) and viability (b), volume of nectar (c), sugar concentration (d), inflorescences (e), floral buds (f) and flowers per day (g) between *Qualea multiflora* and *Q. parviflora*. Significance: ***: p<0.001; ns: p<0.05. The figure shows the raw data, the boxplot delimited by the mean, and the Kernel density estimate graph.

Table S2. Circular statistics considering the flowering of *Qualea multiflora* and *Q. parviflora* from March/2019 to February/2020.

	Mean vector (μ)	*Month*	*Mean vector length (r)*	*Median*	*Circular standard deviation*	*Rayleing test (Z)*	*Rayleing test (p)*	*Watson-two test U^2 (p)*
Q. multiflora	272.8	December	0.916	269.9	24.033	0.916	<0.001	1.721 (<0.001)
Q. parviflora	196.7	September	0.966	210.0	15.029	0.966	<0.001	
